# CRISPR-Cas9 Causes Chromosomal Instability and Rearrangements in Cancer Cell Lines, Detectable by Cytogenetic Methods

**DOI:** 10.1089/crispr.2019.0006

**Published:** 2019-12-16

**Authors:** Emily Rayner, Mary-Anne Durin, Rachael Thomas, Daniela Moralli, Sean M. O'Cathail, Ian Tomlinson, Catherine M. Green, Annabelle Lewis

**Affiliations:** ^1^ Cancer Genetics and Evolution Laboratory, Institute of Cancer and Genomic Sciences, College of Medical and Dental Sciences, University of Birmingham, Birmingham, United Kingdom; University of Oxford, Oxford, United Kingdom.; ^2^ Chromosome Dynamics Core, University of Oxford, Oxford, United Kingdom.; ^3^ Cancer Gene Regulation Group, Wellcome Centre for Human Genetics, University of Oxford, Oxford, United Kingdom; ^4^ Oxford Institute of Radiation Oncology, University of Oxford, Oxford, United Kingdom.

## Abstract

CRISPR-Cas9 has quickly become the method of choice for genome editing, with multiple publications describing technical advances and novel applications. It has been widely adopted as a tool for basic research and has significant translational and clinical potential. However, its usage has outpaced the establishment of essential and rigorous controls for unwanted off-target effects, manifested as small mutations, large deletions of target loci, or large-scale chromosomal rearrangements. A common application of CRISPR-Cas9 is as a tool for creating isogenic cell-line models to study the effects of precise mutations, or variants, on disease traits. Here, we describe the effect of standard CRISPR-Cas9 mutagenesis protocols on well characterized cancer cell lines. We demonstrate that commonly used methods for detecting correctly mutated clones fail to uncover large-scale rearrangements. We show that simple cytogenetic methods can be used to identify clones carrying chromosomal abnormalities and large mutations at target loci. These methods are quick and cost-efficient, and we suggest that such controls should be performed prior to publication of studies based on novel CRISPR-Cas9 mutated cancer cell lines.

## Introduction

Since the landmark publications by the Doudna, Charpentier, Siksnys, Zhang, Joung, and Church labs in 2013, CRISPR-Cas9 has utterly revolutionized the field of biology.^1^ There are many reasons why the adoption of the technology has been so swift: its ease of use, low cost, flexibility, and, undoubtedly, its immediate and obvious application to so many current scientific questions. The potential clinical applications of CRISPR-Cas9 are many and varied, although the ethical questions they raise are complicated.^2,3^

However, like any novel technique, CRIPSR-Cas9 has imperfections, most importantly its propensity to cause off-target mutations, both small and large scale. Authors and reviewers have sometimes neglected the need for rigorous screening for such errors, and this must be addressed before the technique can be relied upon and advanced to the clinic. From the early stages of its development, researchers have identified the ability of the CRISPR-Cas9 machinery to tolerate mismatches in the guide sequence.^4,5^ Improvements in synthetic guide RNA (sgRNA) design algorithms, the modified Cas9 nickase system that utilizes two guide sequences to increase specificity, and sequencing of all predicted off-target regions in correctly mutated cells all aim to reduce mutations at loci with homology to selected guide sequences.^6^ However, more recently, there has been a series of publications describing large-scale deletions at or near the target loci.^7,8^ This type of undesired effect is much harder to avoid and can go undetected using standard screening methods. Kosicki *et al*. and Shin *et al*. used long-range polymerase chain reactions (PCRs) and long-read sequencing technologies to identify the changes. While such sequencing technologies are powerful, they are not yet universally available. Interestingly, Paulis *et al*.^9^ reported a fluorescence *in situ* hybridization (FISH)-based method to detect off-target donor plasmid integrations in CRISPR-Cas9 targeted mouse embryonic stem cells. They noted that these integrations occurred frequently but rarely at predicted off-target loci, and suggested that the integrations occurred at the sites of spontaneous double-strand breaks rather than those generated by Cas9. Ideally, therefore, CRISPR-Cas9 clones required for downstream analysis should be tested for all types of off-target events by whole-genome sequencing. Clearly, quicker pre-screening methods are desirable so that only a few clones need to be checked by this relatively high-cost and analytically intensive technique.

We and others are interested in using CRISPR-Cas9 to mutate well-characterized cancer cell lines in order to generate isogenic control and test cell lines to investigate mutations and variants associated with colorectal cancer (CRC) predisposition or treatment resistance. One of the major goals of creating isogenic cell lines by genome editing is to generate highly specific mutations in endogenous loci without needing to introduce selectable markers or additional insertions such as loxP sites. These cell lines can then be used to study the mechanistic effects of commonly occurring cancer mutations, risk variants, or combinations thereof, and ultimately as tools for developing personalized therapeutics. If there are unexpected and uncharacterized differences between parental and mutated cells, downstream analyses can easily give rise to flawed or misinterpreted results. Cancer cell lines have increased levels of genomic instability when compared to the primary cells and cell lines with stable karyotypes that have been used in the development of CRISPR methodologies. The effects of CRISPR-Cas9 mutagenesis on chromosomally unstable cells has not been fully investigated, heightening the importance of controlling for off-target or undesired mutations.

Here, we describe a number of different CRISPR-Cas9 mutation experiments on cancer cell lines. We identified clones carrying desired mutations according to standard Sanger sequencing in all cases. However, cytogenetic analyses including karyotyping and locus-specific FISH revealed widespread genomic instability in some correctly targeted clones. In addition, FISH revealed large-scale deletions and disruptions of the targeted locus that were undetectable using screening PCRs and Sanger sequencing. These rearrangements are specific to individual clones and vary between cell lines with different levels of chromosomal instability (CIN).

## Methods

### Cell lines and cell culture

COLO320 cells were grown in RPMI-1640, and HCC2998 and SW1463 cells were grown in Dulbecco's modified Eagle's medium, supplemented with 10% fetal bovine serum and 1% penicillin streptomycin (Sigma–Aldrich) at 37°C in 5% CO_2_. Cells were regularly tested for mycoplasma contamination. HCT116 wild type and HCT116^E79K^ cells were purchased from Dharmacon Horizon Discovery.

### CRISPR-Cas9 mutation

sgRNA templates to mutate the MLH1 and POLE loci were designed using the Zhang laboratory online tool (see Supplementary Table S1 for sequences), and single-stranded DNA oligos were synthesized. These were annealed and cloned into CRISPR-Cas9 vectors containing wild-type spCas (px330) and Cas9 nickase (px355) previously modified to contain puromycin selectable markers (kind gifts from Dr. Ben Davies, Wellcome Centre for Human Genetics, University of Oxford, Oxford, United Kingdom) according to online protocols (https://www.addgene.org/crispr/zhang/). Homology directed repair (HDR) templates, designed as single-stranded oligos complementary to the sgRNA, contained the desired sequence change and 70 bp homology arms (Eurogentec; Supplementary Table S2). Predesigned Edit-R sgRNAs to mutate NFE2L2, tracrRNAs, and hCMV-PuroR-Cas9 expression plasmid were purchased from Dharmacon Horizon Discovery. Plasmids and RNAs were transfected into the cell lines using Lipofectamine 2000 (Invitrogen) or Dharmafect (Dharmacon) according to the manufacturers' instructions. Puromycin was added 24 h post transfection at concentrations from 1 to 10 μg/mL and replaced daily until all control green fluorescent protein (GFP) transfected cells were killed. CRISPR-Cas9 transfected cells were then diluted to a concentration of 1 cell per well and the resultant single-cell clones amplified, replica plated, and DNA extracted for screening.

### Cas9 ribonuclear protein delivery

SgRNAs were synthesized using an Engen sgRNA synthesis kit (New England Biolabs) according to the manufacturer's instructions and purified using Zymo-Spin™ IC Columns (Zymo Research; see Supplementary Table S1 for sequences). A non-targeting control sg-RNA supplied with the Engen kit uses a sequence from the tetR(C) gene in the pBR322 plasmid that is not found in the human genome. The most similar human sequence has three mismatches. RNP complexes with Cas9 protein (New England Biolabs) and synthesized sgRNAs were made and mixed with HDR template (Supplementary Table S2) immediately prior to transfection by electroporation with an Amaxa 4D-Nucleofector (Lonza), carried out using program CM-150 and SG cell line kit (Lonza) according to the manufacturer's instructions. Forty-eight hours post transfection, cells were diluted to a concentration of 1 cell per well and the resultant single-cell clones amplified, replica plated, and DNA extracted for screening.

### Mutation screening by PCR and Sanger sequencing

PCR was used to amplify the target loci (see Supplementary Table S1 for primers). Purified PCR products were sequenced by standard Sanger technology (Zoology Sequencing facility, University of Oxford). Sequences were analyzed for quality of trace, aligned to reference sequences with the open source “A plasmid Editor” (ApE v2.0.55), and manually inspected for desired and undesired sequence changes by two independent researchers.

### Preparation of metaphase spreads and DAPI staining

Chromosome spreads were harvested using standard techniques. Briefly, Colcemid (Thermo Fisher Scientific) was added to subconfluent cultures at a final concentration of 50 ng/mL for 3 h. Metaphases were detached by mild trypsinization and swollen in hypotonic solution (KCl 75 mM) before being fixed twice in Carnoy's fixative. Twenty microliters of the cell suspension was dropped onto clean slides and allowed to dry overnight. The slides were mounted in DAPI/Vectashield (Vector Laboratories). Images were collected with an Olympus BX-51 microscope, equipped with a JAI CVM4+ CCD camera, using Leica Cytovision Genus v7.1. Chromosome number was analyzed in a minimum of 25 metaphases per cell line.

### FISH

BAC probes that were not available in-house were purchased from SourceBioscience. The plasmids containing chromosome-specific centromeric DNA were a kind gift from Prof. Mariano Rocchi (Bari University, Bari, Italy). The probes were labeled with the Nick Translation Kit (Abbott Molecular) according to the manufacturer's instructions, with biotinylated-16-dUTP (Sigma–Aldrich), Spectrum Red-dUTP (Vysis, Abbott Molecular), Spectrum-Green dUTP (Vysis, Abbott Molecular), and Spectrum Gold-dUTP (Enzo Laboratories). The probes were purified by precipitation, adding a 10 × excess of unlabeled Cot1 DNA (Thermo Fisher Scientific), and re-suspended in hybridization buffer (50% formamide, 10% dextran sulfate, 2 × SSC). The labeled probes were denatured for 8 min at 85°C in a thermocycler machine. The BAC/PAC probes were pre-annealed at 37°C for 30 min.

Metaphase spread DNA was denatured in NaOH, 0.07 M, for 2 min. Following dehydration in an alcohol series, the denatured probe mix was applied to the slides under a coverslip. The hybridization was carried out overnight at 37°C. Post-hybridization washes were carried out in 0.1 × SSC at 60°C. The biotinylated probes were detected using streptavidin-Cy5 (Thermo Fisher Scientific). The slides were mounted in DAPI/Vectashield (Vector Laboratories) and analyzed with the system described above.

The list of the probes used in this study is shown in Supplementary Table S3.

## Results

**FIG. 1.**
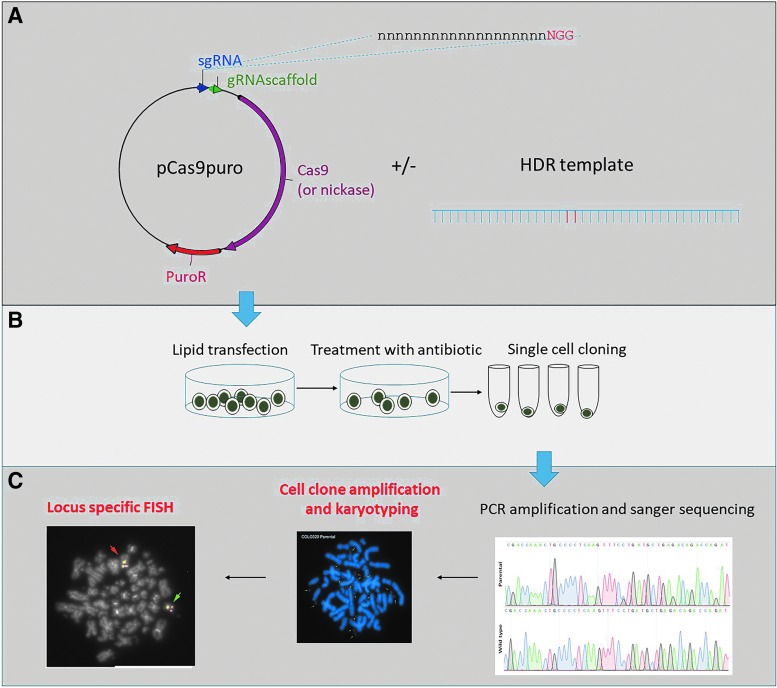
CRIPSR-Cas9 mutation of cell lines: Experimental strategy. Schematic showing **(A)** CRISPR-Cas9 mutation design, **(B)** cell transfection and selection, and **(C)** mutation screening by Sanger sequencing and cytogenetic clone analysis

We carried out CRISPR-Cas9 mutation on three target loci using a different CRC cell line for each (Supplementary Table S1) according to standard protocols (Fig. 1). The experiments were designed to address questions on the theme of CRC genetics and therapy. Experiment 1 aimed to introduce a specific point mutation (by HDR) to mutate a heterozygous single nucleotide polymorphism (SNP) rs1800734 in the *MLH1* promoter. Experiment 2 aimed to revert a pathogenic point mutation in the *POLE* gene to the wild-type sequence by HDR. Experiment 3 aimed to knock out one copy of the *NFE2L2* gene by causing indels in exon 4. The cell lines (COLO320, HCC2998, and SW1463) were selected from publically available resources according to their SNP genotype or mutation status at our loci of interest. Experiments 1 and 2 used Cas9 wild-type and nickase plasmids, respectively, containing sgRNA templates and puromycin selectable markers. Experiment 3 used a puromycin selectable wild-type Cas9 plasmid together with synthetic ready-to-use sgRNAs in an attempt to improve efficiency. For all experiments, the CRISPR-Cas9 machinery was transfected into cells using lipid based methods, followed by puromycin selection and single-cell cloning. Clones were then split into replica plates, with one kept in culture and DNA extracted from the other for mutation screening.

### CRISPR-Cas9 mutated clones were detected by Sanger sequencing

**FIG. 2.**
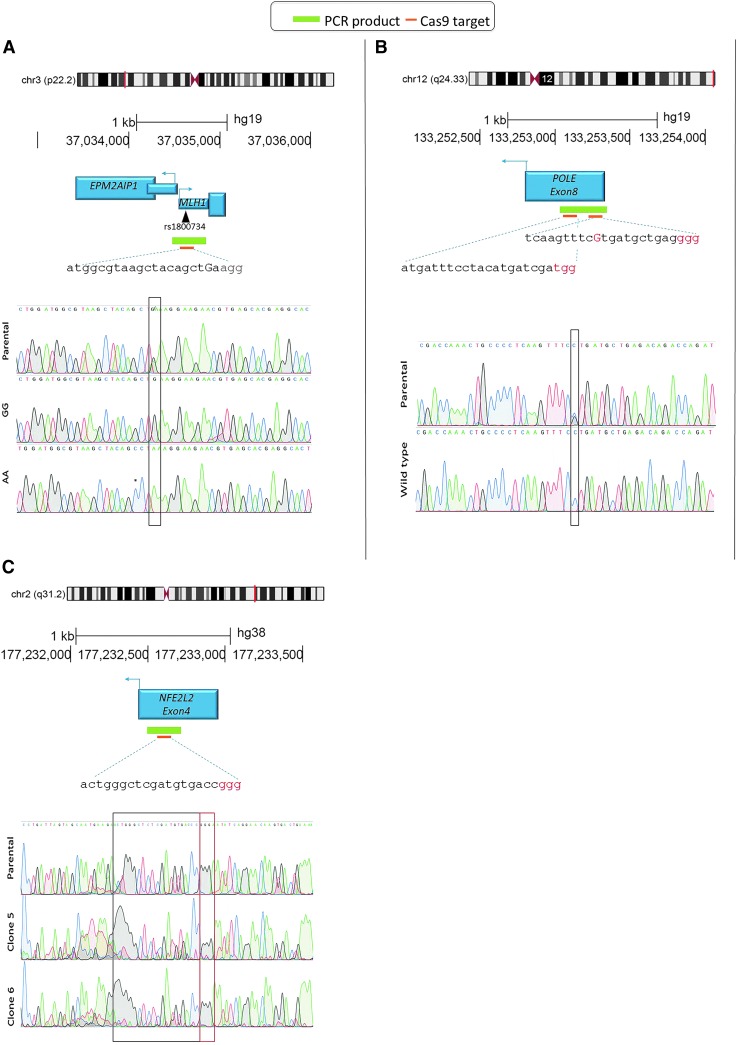
CRISPR target loci and Sanger traces showing expected mutations. **(A)** Mutation of single nucleotide polymorphism (SNP) rs1800734 in the MLH1 promoter in COLO320 cells. The top panel shows the genomic location, target sequence (orange rectangle), and screening polymerase chain reaction (PCR) amplicon (green rectangle). The lower panel shows aligned sequence traces of the parental and two CRISPR-Cas9 mutated clones. The box shows the position of the heterozygous (A/G) SNP location and an AA and GG homozygous trace. **(B)** Reversion of mutation in POLE exon 9 in HCC2998 cells. The top panel shows the genomic location, and two target sequences due to the double-nicking strategy (orange rectangles) and screening PCR amplicon (green rectangle). The lower panel shows aligned sequence traces of the parental and a wild-type revertant clone. The box shows the position of the heterozygous (C to G) mutation location and a homozygous C (wild type) trace. **(C)** Knockout of NFE2L2 exon 4 in SW1463 cells. The top panel shows the genomic location, target sequence (orange rectangle), and screening PCR amplicon (green rectangle). The lower panel shows aligned sequence traces of the parental and clones with heterozygous deletions obtained from the reverse sequencing primer (right to left). The box shows the position of the guide and PAM sequence, within which the clean trace becomes disrupted due to a deletion.

Figure 2A shows sequence traces from experiment 1, suggesting that we correctly mutated the heterozygous A/G SNP creating both GG and AA homozygous clones. Clones with correct sequences were found with a frequency of 1.5% (GG) and 2% (AA). In experiment 2, where the double nicking strategy was used,^6^ correctly targeted point mutations to revert an existing heterozygous mutation to the wild type were found using Sanger sequencing with an efficiency of 9.5% at the POLE locus (Fig. 2B). Heterozygous indels in the *NFE2L2* gene were detected in SW1463 cells at an efficiency of 2% (Fig. 2C). PCR primers were designed to be outside any regions of homology in HDR templates but to give products easily sequenced by a single Sanger read covering 200–300 bp, and centered on the sgRNA template sequence.

### Chromosomal analysis showed differences between CRISPR-Cas9 clones and parental cells

**FIG. 3.**
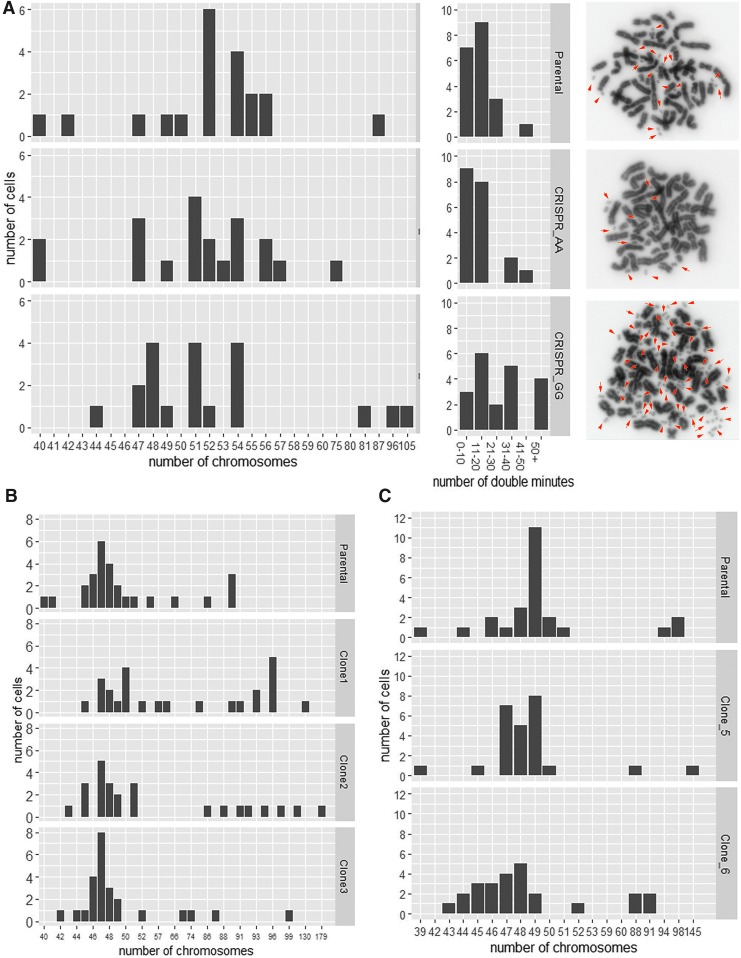
CRIPSR clones exhibit chromosomal instability. **(A)** Mutation of SNP rs1800734 in the MLH1 promoter in COLO320 cells. Left panel: graphs showing the chromosome counts per cell of the parental and AA and GG sequenced clones. The parental cells show a variable number of chromosomes with the modal number of 52. The mutant clones show a wider distribution and no clear model number. Middle panel: graphs showing the number of double minutes per cell. The distribution is similar between the parental and AA clones but numbers are greatly increased in GG cells. Left panel: DAPI-stained metaphase spreads showing double minutes (red arrows). **(B)** Reversion of mutation in POLE exon 9 in HCC2998 cells. Graphs showing the chromosome counts per cell of the parental and clones 1, 2, and 3. The parental cells have a modal number of 47 chromosomes. Clones 2 and 3 also have a modal number of 47 and similar overall distribution. Clone 1 has more variable chromosome numbers. **(C)** Mutation of *NFE2L2* gene in SW1463 cells. Graphs showing the chromosome counts per cell of the parental and sequenced clones 5 and 6. The parental cells show a variable number of chromosomes with the modal number of 49. The mutant clones show a wider distribution and no clear modal number.

Clones with the desired mutations were amplified, and metaphase spreads were subjected to cytogenetic analysis using DAPI staining. We found substantial differences in chromosome numbers between parental cells and some CRISPR-Cas9 clones in all three experiments (Fig. 3), with an increased variability found in the CRISPR-Cas9 clones. In the COLO320 clones, we also observed an increase in the number of double minutes, small fragments of extrachromosomal DNA, as an additional manifestation of CIN in clone GG (Fig. 3A, right panel). While COLO320 and SW1463 (Fig. 3C), like many cancer cell lines, exhibit aneuploidy, HCC2998 is close to diploid. In the latter, we observed that CRIPSR-Cas9 targeted clones 2 and 3 (Fig. 3B) had the same modal chromosomal number and very similar overall counts to the parental cell lines. This suggests that the underlying instability of the cell line acts together with the CRISPR-Cas9 machinery to drive large-scale chromosomal rearrangements. We confirmed this by analyzing a commercially available CRISPR-Cas9 mutated clone of the diploid CIN cell line Hct116. Similar to HCC2998, no large differences in chromosome number or karyotype were observed between the parental and CRISPR-Cas9 mutated clone (Supplementary Fig. S1). It is also possible that the single-cell cloning process exacerbated existing CIN, selecting for clones with rearrangements that enhanced growth under less favorable conditions. In order to check that the subcloning process alone is not responsible for all the observed rearrangements, we subjected COLO320 to single-cell cloning in the absence of CRISPR-Cas9 machinery and analyzed metaphase spreads from five separate clones (Supplementary Fig. S2A and B). Surprisingly, although each clone was derived from a single cell, there were already substantial variations between cells after fewer than 10 passages. However, the distribution of chromosome number and double minutes was similar to that seen in the parental cell population (Fig. 3A). We also wished to confirm that puromycin treatment alone is not the main factor in the increased CIN. We therefore treated COLO320 cells with a near lethal dose of puromycin (resulting in 80–90% cell death), followed by single-cell cloning and chromosome counting. Again, we found that though the cells varied in chromosome number, the distribution was more similar to the parental cell population than those subjected to CRISPR mutagenesis (Supplementary Fig. S2D).

### FISH analysis showed large-scale target locus-specific rearrangements

**FIG. 4.**
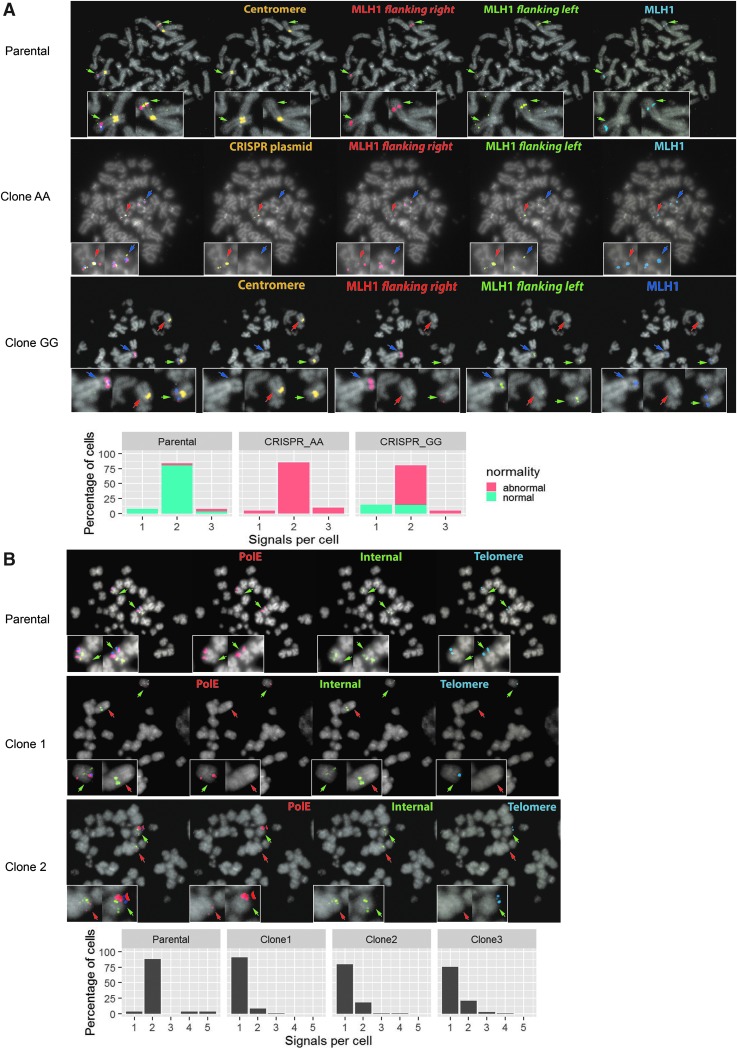
Fluorescence *in situ* hybridization (FISH) showing abnormal signals at the target loci. **(A)** Mutation of SNP rs1800734 in the MLH1 promoter in COLO320 cells. The top row shows signals from the parental cell line with clones apparently mutated to AA and GG below. The far-left panel of each row shows the merged signals, followed by a panel with the chromosome 3 centromere (parental and clone GG) or CRISPR plasmid px330puro (clone AA) labeled in yellow, two probes flanking *MLH1* (RP11-331G2, red; RP11-56P22, green), and a probe binding directly to the locus (RP11-491D6, blue). Green arrows indicate the position of normal signals, red abnormal, and blue correct MLH1 signals on an abnormal chromosome. Magnifications (2 × ) of each signal are embedded into every panel. The graph shows the percentage of cells carrying one, two, or three signals and the proportion of abnormal signals in each category. All CRISPR AA cells contain at least one abnormal signal containing the plasmid backbone. Most CRISPR GG cells have two normal MLH1 loci but only one co-labeling with the chromosome 3 centromere. **(B)** Reversion of mutation in POLE exon 9 in HCC2998 cells. The top row shows signals from the parental cell line with clones 1 and 2 with apparent reversion to wild-type sequence shown below. The far-left panel of each row shows the merged signals, followed by a probes binding to the *POLE* locus (RP11-148L11, red), internal between *POLE* and the centromere (RP11-25J3, green), and telomeric to *POLE* (CTC221K18, blue). Green arrows indicate the position of normal signals, red abnormal. Magnifications (2 × ) of each signal are embedded into every panel. The graph shows the percentage of cells carrying one to five normal *POLE* signals per cell in the parental and clones 1 and 2. In clone 1, only one normal signal is seen in the majority of cells. In clone 2, one normal is seen and one with reduced *POLE* and no telomeric signal, suggestive of a truncation breakpoint within the *POLE* probe binding region.

Given the observed combination of background and CRISPR-Cas9 induced CIN, we also wished to investigate any rearrangements within the target locus more directly attributable to the CRISPR-Cas9 targeting. We therefore carried out FISH on mutated clones from each experiment using two or three BAC probes overlapping and flanking the target locus, together with centromeric or telomeric probes for the appropriate chromosome (Supplementary Table S3). Figure 4A shows the FISH analysis of the parental and CRISPR-Cas9 mutated COLO320 cells from experiment 1. We detected signals on both copies of chromosome 3 on the majority of parental cells, comprising all three *MLH1* BAC probes, and therefore exhibiting the expected labeling pattern for the diploid *MLH1* locus. In contrast, the AA clone had no cells containing two correct signals. All cells had one correct signal and one containing the three BAC probes plus additional labeling with the centromeric probe overlapping the *MLH1* locus (Supplementary Fig. S3). Using a labeled version of the CRISPR-Cas9 px330 vector in a further FISH experiment, we confirmed that the signal was in fact due to integration of this vector, probably in a tandem array, hybridizing to the generic vector sequence present in the centromeric probe backbone (Fig. 4A). Disruption of our target by a large vector insert likely explained the disappearance of one of the two SNP alleles within the Sanger sequence (giving the appearance of an AA homozygote), since the screening PCR primers failed to amplify across such a large insertion. The GG clone showed two normal MLH1 signals, although one had translocated in its entirety to a different unidentified chromosome. The translocation could have happened as a result of a double-strand break caused by Cas9 or may have been present in the parental cell prior to mutation at very low frequency. Subclones that had not undergone CRISPR-Cas9 treatment had very few abnormal FISH signals, with most cells having two complete MLH1 loci on chromosome 3 (Supplementary Fig. S2C).

Further FISH analysis of HCC2998 clones from experiment 2 demonstrated that apparent CRISPR-Cas9 point mutations correcting heterozygous POLE mutations were in fact due to chromosomal truncations in which the mutated copy of POLE is lost (Fig. 4B). In clone 1 (and clone 3; data not shown), we observed one normal POLE locus signal and total loss of the other, whereas in clone 2, one of the two POLE signals was much weaker, suggesting partial truncation of the target region due to deletion within the probe binding site. In all clones, we suggest that the cellular machinery failed to repair a double-strand break at POLE caused by the CRIPSR-Cas9 machinery. Again, PCR amplification would have failed to amplify this truncated locus, giving rise to Sanger sequence from just one allele and the appearance of homozygosity. Similar truncations were seen in a separate cell line, SNU81 after CRISPR-Cas9 POLE targeting (data not shown), so the phenomenon is not cell-type specific. FISH on clones from experiment 3 showed frequent fragmentation of chromosome 2 in SW1463 cells (data not shown).

The use of purified Cas9 ribonuclear protein (RNP) delivery methods has been shown to be more efficient and lead to fewer off-target events by several groups.^10–13^ We therefore repeated experiment 1 using this approach. Initially, we were encouraged that RNP appeared more efficient than plasmid-based methods (even without antibiotic selection) after screening by Sanger sequencing (6% AA, 4% GG). However, when we subjected these clones, and other clones showing heterozygous inserts or deletions in the region to karyotyping and FISH analysis, we again found abnormal chromosome counts and FISH signals, suggesting that even with a short exposure to Cas9 protein, cancer cells are prone to large rearrangements (Supplementary Figs. S4 and S5 and Supplementary Table S4). A clone genotyped as AA was polyploid, with most cells carrying four or more copies of *MLH1* (Supplementary Fig. S4A), and a clone genotyped as GG had lost one copy of *MLH1* and a flanking region in all cells analyzed (Supplementary Fig. S4B). The clones treated with an off-target sgRNA had largely normal *MLH1* loci, as seen in the parental cells, confirming that the targeted Cas9 sgRNA complex is crucial for generating large-scale rearrangements at the target locus. However, these cells also displayed more tetraploidy and variable chromosome counts than untreated clones, confirming that the action of Cas9, even without a target, can augment the underlying CIN of the cells (Supplementary Table S4 and Fig. S5).

## Discussion

We have shown here that standard CRISPR-Cas9 genome editing protocols in cancer cell lines with existing CIN are highly likely to cause unwanted chromosomal rearrangements both at the target loci and on other chromosomes. We were able to detect these events using cytogenetic analysis. We specifically used relatively straightforward methods to demonstrate that rearrangements are easy to detect without specialist resources or expertise. We used our in-house facility, but there are similar commercial services readily available. In future experiments in our own lab, we will now be carrying out routine analysis to select a subclone of the parental cell line prior to carrying out CRISPR-Cas9 mutagenesis, and afterwards on clones with apparently correct mutations according to Sanger or next-generation sequencing (NGS).

Due to their utility in investigating cancer biology and therapeutic responses, gene-edited cancer cell lines are highly desirable.^14,15^ Clinical adoption of NGS means that mutations can be quickly and precisely identified in individual cancer patients, and transferring this genetic information into cell lines using CRISPR-Cas9 will create models in which to develop highly personalized therapies. We found that diploid cancer cells were more likely to maintain their karyotype during the CRISPR-Cas9 process, whereas clones from aneuploid parental cell lines showed high levels of instability due to a combination of their underlying CIN and selection during the single-cell cloning process.

This study focused on cancer cell lines that are inherently more unstable than primary cells and whole model organisms. We have not carried out an in-house comparison using our methods on primary cells, and therefore our conclusions here are limited to cancer cell lines. It is likely that karyotypic and aneuploid effects will prove less important in existing and novel CRISPR-Cas9 models with stable genomes such as transgenic mice. However, chromosomal truncations have recently been reported after CRISPR-Cas9 in HEK293 cells also detected by FISH.^16^ The authors found that using a single nickase approach reduced the likelihood of truncations, which presumably arise after failure to repair double-strand breaks. In addition, large insertions and deletions in or near the target locus have been previously reported in mice, mouse embryonic stem cells, and human differentiated cells.^7,8^ These authors used long-range PCRs and long-read sequencing spanning up to 30 kb of the target locus to detect large mutations. Both techniques have their advantages, and best practice might be to use them in combination before biological analysis of the final selected clone or transgenic line. Small deletions outside BAC probes will be more easily detected using sequencing, whereas large deletions >30 kb, chromosome truncations, and translocations may only be observed using FISH.

The detrimental effects of CIN and large deletions may vary according to the nature of the planned downstream analysis. The fact that many mutations and cancer-associated variants occur in genes related to genomic integrity adds additional confounding effects, meaning that the utility of each clone must be considered on a case-by-case basis. For instance, in experiment 1, we wished to observe the effect of mutating a promoter SNP on transcriptional activation of the adjacent MLH1 promoter *in cis*. FISH analysis of clone GG showed an intact *MLH1* locus, albeit translocated to a different chromosome, making carefully controlled localized *in cis* analysis possible. In experiment 2, we aimed to revert an oncogenic mutation to wild type but instead deleted the entire oncogenic copy of POLE. Thus, the end result was still the removal of the mutation and a hemizygous wild-type clone. Since genes distal to the breakpoint (Supplementary Table S5) were found to be unlikely to affect replication or repair, and loss of one copy of POLE does not predispose to cancer,^17^ selected experiments were possible on these cell lines. However, in experiment 3, we wished to assess the effect of reducing NRF2 expression on resistance to chemo- and radiotherapies. In this case, the observed karyotypic changes will have far greater effects on the response to DNA damaging therapies than the intended modest reduction of NRF2 activity.

We propose that novel CRISPR-Cas9 mutated cell lines or other model systems should only be published with appropriate combinations of sequencing and cytogenetic controls for off-target effects. Simply sequencing off-target regions predicted by algorithms is not sufficient. Cryptic off-target mutations have been shown to occur at regions of DNA “stretching,” with up to 10 mismatches to the original guide sequence,^18^ making it virtually impossible to rule out undesired mutations. It should therefore become standard to use multiple clones or demonstrate the causality of the mutation using reversion experiments in order to confirm that any phenotypic differences are solely due to targeted mutations.

There are continual advances in CRISPR-Cas9 methodology, and some of these will certainly reduce unwanted mutations. For example, Cas9 ribonuclear proteins (RNP) are now routinely used instead of plasmid vectors for transfection of the CRIPSR machinery into cells.^10,12^ This route increases efficiency, results in a more transient Cas9 activity, and will prevent the integration of vector sequences such as we observed. However, this approach does not prevent chromosomal rearrangements, as we have demonstrated. Re-engineering of the Cas9 protein and modifying the sgRNA scaffold have also been shown to increase target specificity.^19,20^ We expect that more efforts will now be made to limit the instances of large deletions

In summary, we present a cost-effective visual method for assessing chromosomal rearrangements and large deletions in CRISPR-Cas9 mutated clones. We demonstrate that these are frequent and significant events in cancer cell lines, which would have implications for any downstream analysis.

## Supplementary Material

Supplemental data

Supplemental data

Supplemental data

Supplemental data

Supplemental data

Supplemental data
