# Supplementary material for: CRISPR-Cas9 Causes Chromosomal Instability and Rearrangements in Cancer Cell Lines, Detectable by Cytogenetic Methods
Source: CRISPR J. 2019 Dec 16;2(6):406–16. doi: 10.1089/crispr.2019.0006 (PMC6919265; doi:10.1089/crispr.2019.0006)
Supplement: Supplemental data [file Supp_Table_1_2.pdf]

Supplementary Table S1. Details of Experimental Aims, Loci, Cells, Target Sequences, and Screening Primers

Experiment	*Gene*	*Cell Line*	*Target sequence*	*Desired mutation*	*Genomic location*	*Screening primers*	*Size (bp)*
1	MLH1	COLO320, colon, adenocarcinoma, hyperdiploid	ATGGCGTAAGCTACAGCTGA (G to A)	Mutation of heterozygous SNP: rs1800734 A/G to homozygous AA and GG	Chr3: 36993437–36993456	F-AGCAACCCACAGAGTTGAGA	213
			ATGGCGTAAGCTACAGCTAA (A to G)			R-GAATAACCCCTGCCACGAAC	
2	POLE	HCC2998, colon, adenocarcinoma, diploid	TCAAGTTTCgTGATGCTGAG	Revertant of a pathogenic point mutation	Chr12: 133253174–133253193	F-CTAACAGTGGGGCAGATGCT	258
			ATGATTTCCTACATGATCGA		Chr12: 133253139–133253158	R-GGTGTTCAGGGAGGCCTAAT	
3	NFE2L2	SW1463, rectum, adenocarcinoma, hyperdiploid	ACTGGGCTCGATGTGACC	Indel causing gene knockout	Chr2: 177232522–177232544	F-AGGCCAAGTAGTGTGTCTCC; R-ATGCCCTCACCTGCTACCTTT	224
			GTGTTCTGGTGATGCCACAC		Chr2: 177231526–177231548	F-AGGCCAAGTAGTGTGRCTCC; R-ATGCCCTCACCTGCTACTTT	224
			TATTTGACTTCAGTCAGCGA		Chr2: 177234188–177234210	F-TGATTGACATACTTTGGAGGCA; R-GCTGGCTGAATTGGGAGAAA	211

Supplementary Table S2. HDR Template Sequences

Experiment	*Gene*	*HDR template*
1	MLH1 A to G	GACTGGCATTCAAGCTGTCCAATCAATAGCTGCCGCTGAAGGGTGGGGCTGGATGGCGTAAGCTACAGCTgAAGGAAGAACGTGAGCACGAGGCACTGAGGTGATTGGCTGAAGGCACTTCCGTTGAGCATCTAGACGTTT
1	MLH1 G to A	GACTGGCATTCAAGCTGTCCAATCAATAGCTGCCGCTGAAGGGTGGGGCTGGATGGCGTAAGCTACAGCTaAAGGAAGAACGTGAGCACGAGGCACTGAGGTGATTGGCTGAAGGCACTTCCGTTGAGCATCTAGACGTTT
2	POLE	ATTTTGTCCCCACAGGACCCTGTGGTTTTGGCATTTGACATTGAGACGACCAAACTGCCCCTCAAGTTTCcTGATGCTGAGACAGACCAGATTATGATGATTTCCTACATGATCGATGGCCAGGTGAGCAGGTGGCTTCTG
