# Supplementary material for: CRISPR-Cas9 Causes Chromosomal Instability and Rearrangements in Cancer Cell Lines, Detectable by Cytogenetic Methods
Source: CRISPR J. 2019 Dec 16;2(6):406–16. doi: 10.1089/crispr.2019.0006 (PMC6919265; doi:10.1089/crispr.2019.0006)
Supplement: Supplemental data [file Supp_Table_3-5.pdf]

Supplementary Table S3. Details of Probes Used in FISH Experiments

BAC/PAC/plasmid	*Start (hg19)*	*End (hg19)*	*Cytogenetic band*	*Symbol*
RP11-56P22	35,006,192	35,156,133	3p22.3	MLH flanking left
RP11-491D6	37,059,146	37,161,516	3p22.2	MLH
RP11-331G2	39,160,068	39,336,652	3p22.2	MLH flanking right
pAE068	na	na	Chromosome 3 centromere	Cen3
px330	na	na	na	CRISPR Plasmid
RP11-25J3	75,892,543	76,047,496	12q21.2	Chr12 Internal probe
RP11-148L11	133,038,915	133,243,361	12q24.33	PolE
CTC221K18[S1]	na	na	12q24.3	Telo 12q

Supplementary Table S4. Mutation of SNP rs1800734 in the MLH1 Promoter in COLO320 Cells Using RNP Delivery: Summary of FISH Signals at the Target Loci

		Number of MLH1 FISH signals (% of cells)				
Clone and mutation	*Metaphases analyzed*	*1 copy*	*2 copies*	*3 copies*	*4 copies*	*More than 4 copies*
AA targeted indel 1	28	10.70%	75%	—	14.3% (tetraploid)	—
AA targeted indel 2	26	—	88%	4%	8% tetraploid	—
AA targeted indel 3	25	8%	80%	8%	4%	—
AA targeted AA	25	4%	16%	20%	20%	40%
GG targeted indel 1	28	—	96.40%	—	8% (tetraploid)	—
GG targeted indel 2	26	—	92%	—	8% (tetraploid)	—
GG targeted GG	25	88%	12% (tetraploid)	—	—	—
Non-target wild type 1	28	—	96%	—	4% (tetraploid)	—
Non-target wild type 2	25	16%	72%	4%	8% (tetraploid)	
Non-target wild type 3	26	24%	56%	12%	12% (tetraploid)	
Puro treated Clone1	40	7.50%	90%	2.50%	—	—
Puro treated Clone2	25	—	100%	—	—	—

The two clones with the desired mutation according to Sanger sequencing are highlighted in gray. The percentage of clones with two normal FISH signals is conditionally highlighted in shades of green.

Supplementary Table S5. Genes Deleted in Chromosomes Truncated Due to Unrepaired DSBs After CRISPR-Cas9 Mutation at *POLE*

Genes distal to POLE on chr12q
PXMP2
PGAM5
ANKLE2
COLGA3
CHFR
ZNF605
ZNF26
ZNF84
ZNF140
ZNF891
ZNF269
ZNF10

Supplementary Reference

S1. Knight SJ, Lese CM, Precht KS, et al. An optimized set of human telomere clones for studying telomere integrity and architecture. Am J Hum Genet 2000;67:320–332. DOI: 10.1086/302998.
