# Supplementary material for: CRISPR-Cas9 Causes Chromosomal Instability and Rearrangements in Cancer Cell Lines, Detectable by Cytogenetic Methods
Source: CRISPR J. 2019 Dec 16;2(6):406–16. doi: 10.1089/crispr.2019.0006 (PMC6919265; doi:10.1089/crispr.2019.0006)
Supplement: Supplemental data [file Supp_Fig1.pdf]

Supplementary Data

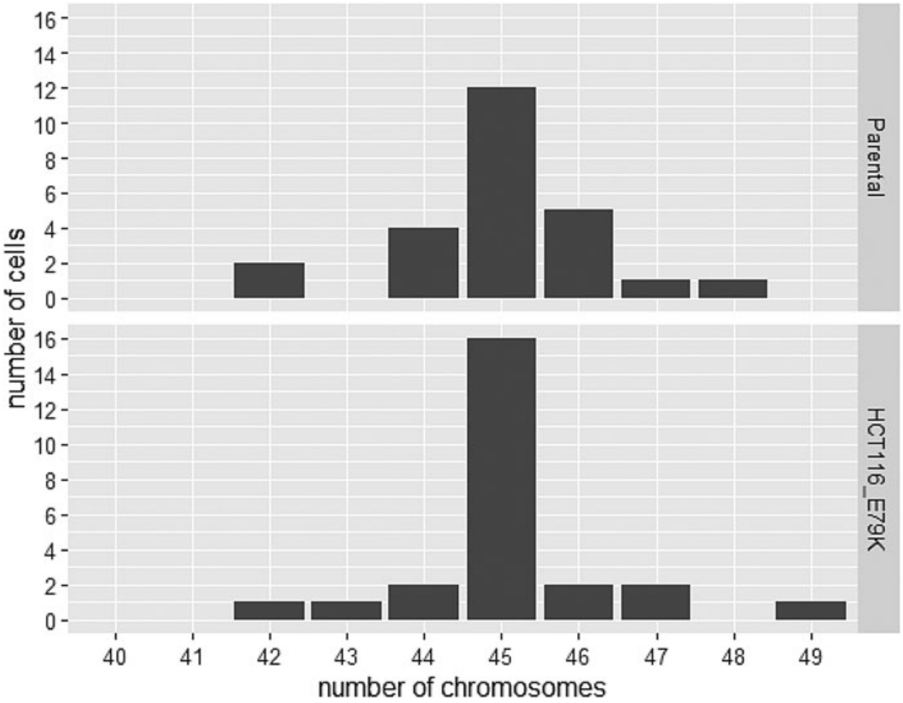

SUPPLEMENTARY FIG. S1. Hct116 CRIPSR clone has a normal chromosome count. Graphs showing the chromosome counts per cell of the parental and E79K mutant line. Both lines have a modal number of 45 chromosomes and a similar distribution.
