# Supplementary material for: CRISPR-Cas9 Causes Chromosomal Instability and Rearrangements in Cancer Cell Lines, Detectable by Cytogenetic Methods
Source: CRISPR J. 2019 Dec 16;2(6):406–16. doi: 10.1089/crispr.2019.0006 (PMC6919265; doi:10.1089/crispr.2019.0006)
Supplement: Supplemental data [file Supp_Fig2.pdf]

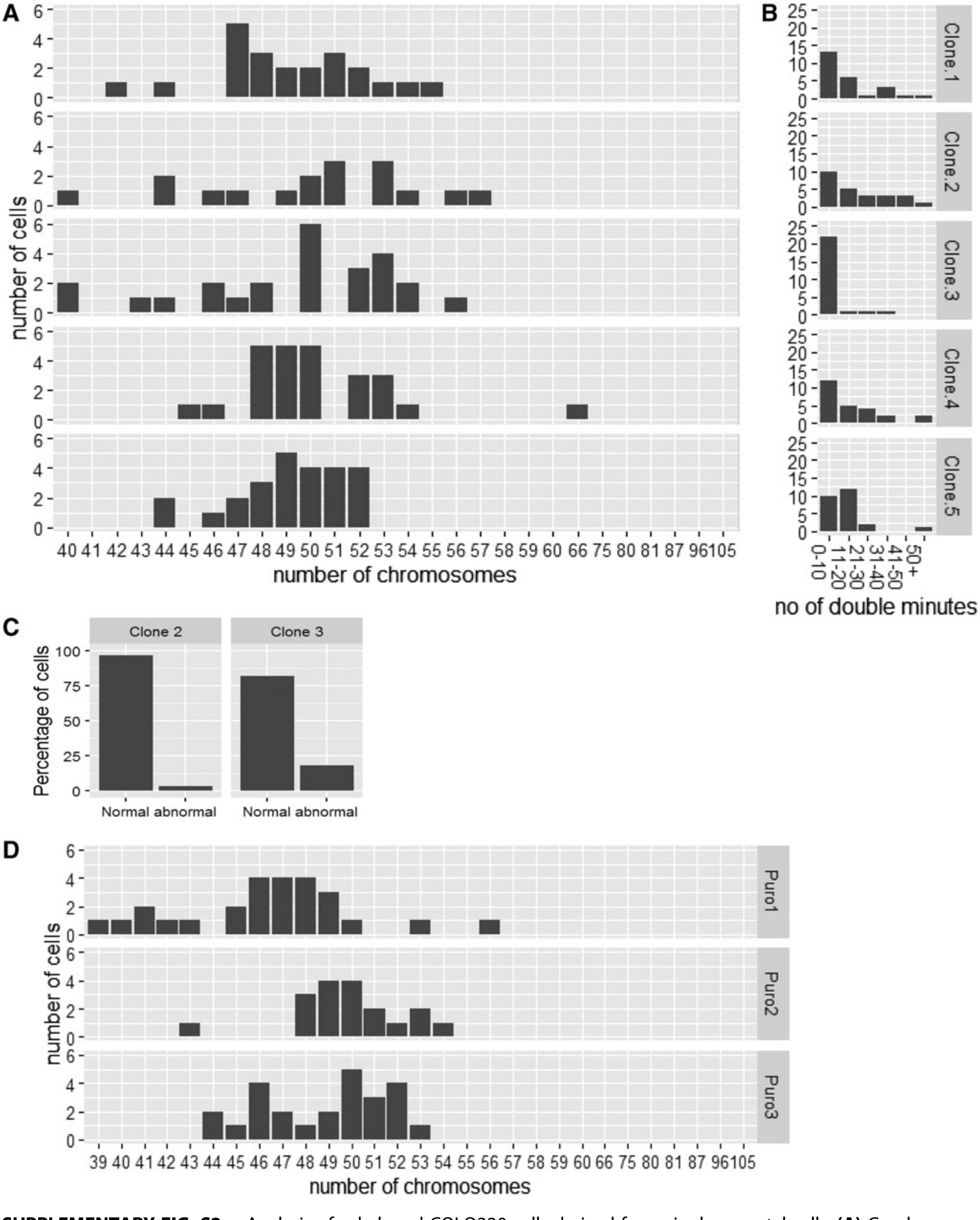

SUPPLEMENTARY FIG. S2. Analysis of subcloned COLO320 cells derived from single parental cells. **(A)** Graphs showing the chromosome counts per cell of five different subclones. The counts are variable but all within the range seen in the parental cell line. **(B)** Numbers of double minutes per cell for each clone. Few clones show more than 20 double minutes, as in the parental cell lines. **(C)** Results of MLH1 FISH. The graph shows the percentage of cells with normal (two MLH1 signals on chromosome 3 per cell) or abnormal (one or three signals per cell, incomplete MLH1 signal or MLH1 on a chromosome other than chromosome 3). **(D)** Graphs showing the chromosome counts per cell of puromycin treated subclones. The counts are variable but all within the range seen in the parental cell line and untreated subclones, suggesting that puromycin has a negligible effect on chromosomal instability.
