# Supplementary material for: CRISPR-Cas9 Causes Chromosomal Instability and Rearrangements in Cancer Cell Lines, Detectable by Cytogenetic Methods
Source: CRISPR J. 2019 Dec 16;2(6):406–16. doi: 10.1089/crispr.2019.0006 (PMC6919265; doi:10.1089/crispr.2019.0006)
Supplement: Supplemental data [file Supp_Figs3_4.pdf]

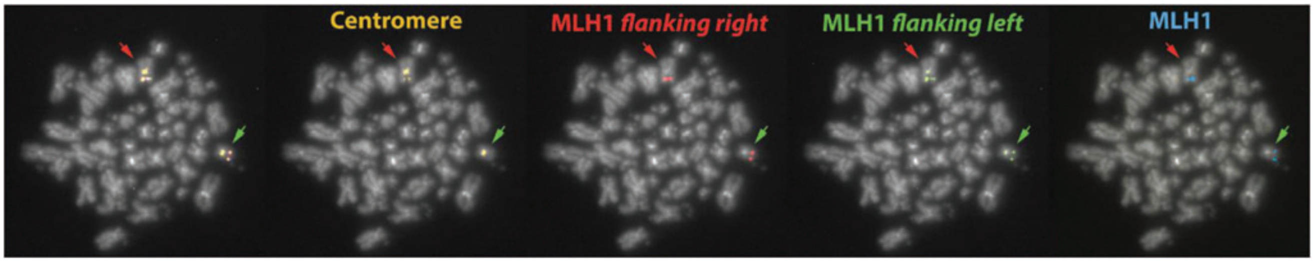

SUPPLEMENTARY FIG. S3. FISH demonstrating the cross-hybridization of centromeric probe with plasmid sequence in clone CRISPR AA. The far-left panel shows the merged signals, followed by a panel with the chromosome 3 centromere (labeled in yellow), two probes flanking *MLH1* (RP11-331G2, red; RP11-56P22, green), and a probe binding directly to the locus (RP11-491D6, blue). Green arrows indicate the position of normal signals and red abnormal with the centromeric probe binding within the *MLH1* locus.

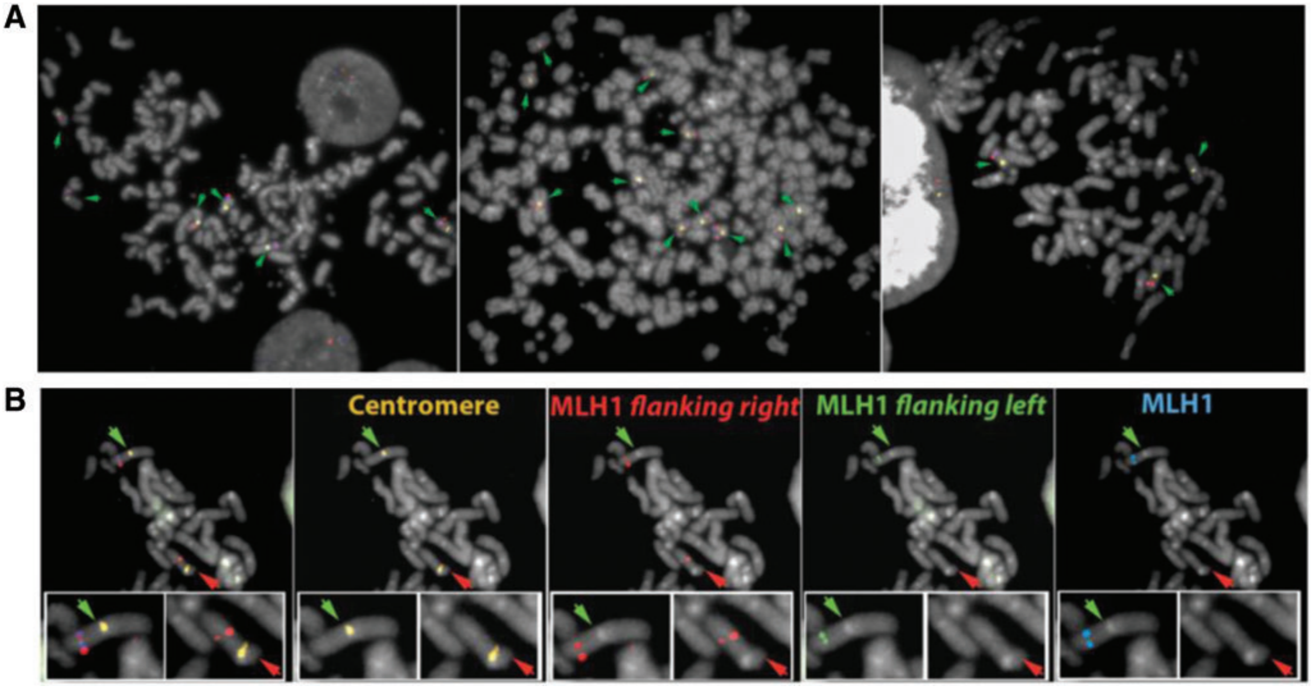

SUPPLEMENTARY FIG. S4. FISH showing abnormal signals at the *MLH1* locus after delivery of CRISPR machinery by RNP. **(A)** Representative pictures of three metaphase spreads from a clone genotyped as AA in which most cells are tetra- or polyploid and show multiple *MLH1* signals (green arrows). Each picture shows the merged signals from: the chromosome 3 centromere (labeled in yellow), a probe flanking *MLH1* (RP11-331G2, red), and a probe binding directly to the locus (RP11-491D6, blue). **(B)** Representative picture of clone genotyped as GG in which most cells have lost one copy of the *MLH1* gene and one flanking region. The far-left panel shows the merged signals, followed by a panel with the chromosome 3 centromere (labeled in yellow), two probes flanking *MLH1* (RP11-331G2, red; RP11-56P22, green), and a probe binding directly to the locus (RP11-491D6, blue). Green arrows indicate the position of normal signals and red abnormal.
