# Supplementary material for: CRISPR-Cas9 Causes Chromosomal Instability and Rearrangements in Cancer Cell Lines, Detectable by Cytogenetic Methods
Source: CRISPR J. 2019 Dec 16;2(6):406–16. doi: 10.1089/crispr.2019.0006 (PMC6919265; doi:10.1089/crispr.2019.0006)
Supplement: Supplemental data [file Supp_Fig5.pdf]

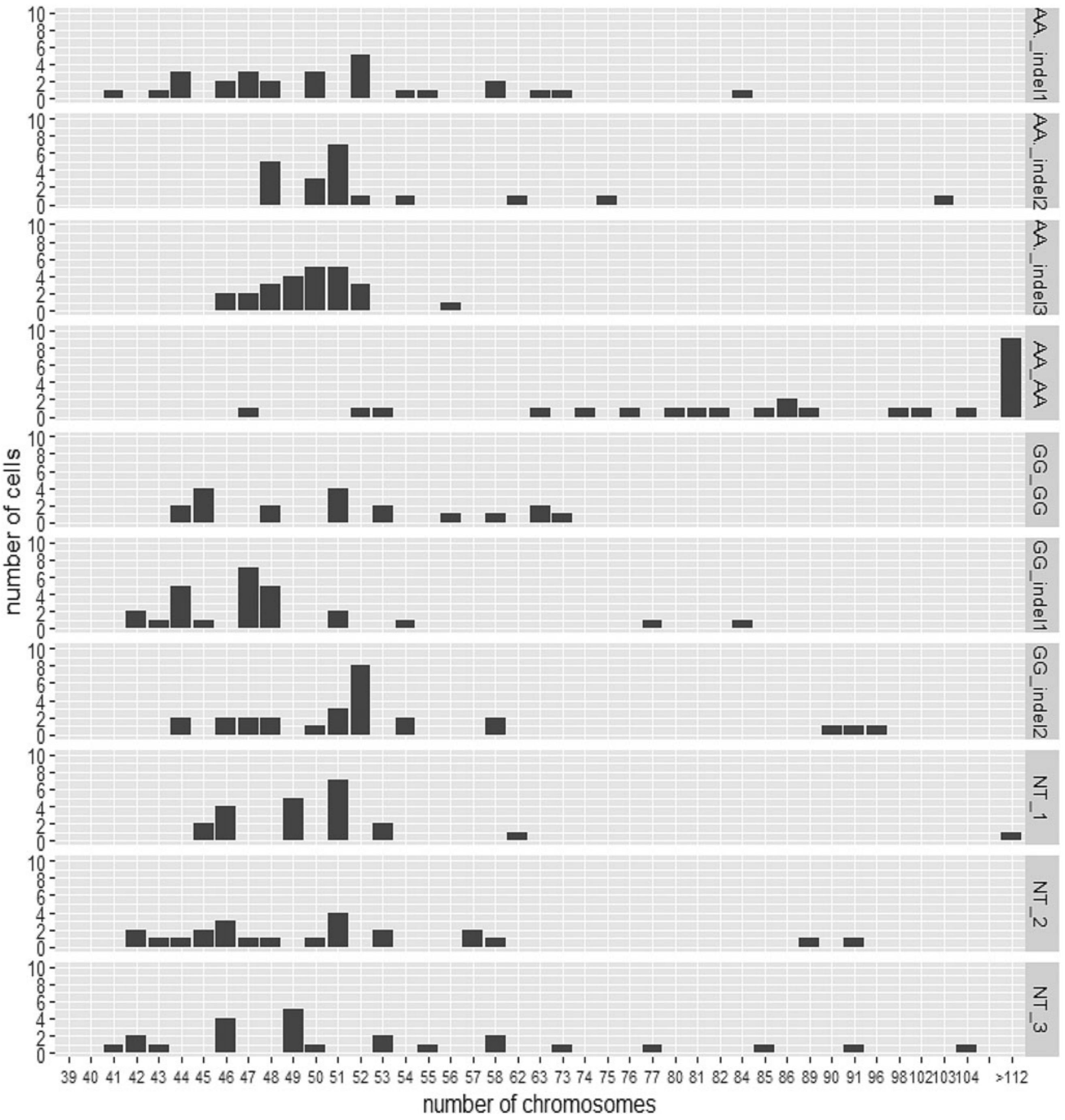

SUPPLEMENTARY FIG. S5. Graphs summarizing the chromosome counts of clones after delivery of CRISPR machinery by RNP to mutate the *MLH* locus. Clones treated with a sgRNA and HDR template designed to generate an AA genotype are labeled AA followed by the mutation according to Sanger sequencing. Clones treated with a sgRNA and HDR template designed to generate a GG genotype are labeled GG followed by the mutation according to Sanger sequencing. Clones treated with non-targeting sgRNA are NT. The targeted and non-targeted clones show a more variable distribution than non-treated controls, sometimes with no clear model number. Clone AA_AA is largely tetraploid.
